# Peripheral Blood Biomarkers Associated With Outcome in Non-small Cell Lung Cancer Patients Treated With Nivolumab and Durvalumab Monotherapy

**DOI:** 10.3389/fonc.2020.00913

**Published:** 2020-06-30

**Authors:** Meilin Jiang, Wenying Peng, Xingxiang Pu, Bolin Chen, Jia Li, Fang Xu, Liyu Liu, Li Xu, Yan Xu, Jun Cao, Qianzhi Wang, Kang Li, Jingyi Wang, Lin Wu

**Affiliations:** The Second Department of Thoracic Oncology, Hunan Cancer Hospital, The Affiliated Cancer Hospital of Xiangya School of Medicine, Central South University, Changsha, China

**Keywords:** non–small cell lung cancer, predictive biomarker, nivolumab, durvalumab, platelet-to-lymphocyte ratio, albumin

## Abstract

**Background:** Selecting patients who potentially benefit from immune checkpoint inhibitors (ICIs) is critical. Programmed death ligand-1 (PD-L1) protein immunohistochemical expression on cancer cells or immune cells and next-generation sequencing-based tumor mutational burden (TMB) are hot spots in studies on ICIs, but there is still confusion in the testing methods. Because blood samples are much easier for clinical application, many potential peripheral biomarkers have been proposed. This study identified blood parameters associated with the outcome of non-small cell lung cancer (NSCLC) patients with ICI monotherapy.

**Materials and Methods:** Data from 76 NSCLC patients were analyzed retrospectively. To assess the connection between survival and peripheral blood markers measured before the first and fifth doses of ICI treatment, we utilized Cox regression model survival analysis and receiver operating characteristic (ROC) curve analysis to assess the markers.

**Results:** In the nivolumab cohort, the optimal cutoffs for predicting 11-month overall survival (OS) were 168.13 and 43 g/L for platelet-to-lymphocyte ratio (PLR) and albumin, respectively. When patients were grouped with PLR and albumin, a significant difference in SD-PR vs. PD rate was found between the high and low groups, which was not found when the patients were grouped by PD-L1 expression. Patients with high PLR (>168.13) or low albumin ( ≤ 43 g/L) before ICI had a significantly increased hazard of progression, separately (for PLR, *P* = 0.006; for albumin, *P* = 0.033), and of death (for PLR, *P* = 0.014; for albumin, *P* = 0.009) compared with those patients who had low PLR or albumin levels. More importantly, we found that a higher PLR (>168.13) before the fifth dose of ICIs was also a prognostic biomarker, which significantly correlated with shorter OS in both the nivolumab (*P* = 0.046) and durvalumab cohorts (*P* = 0.028).

**Conclusions:** PLR and albumin may help in the stratification of high progression and death risk groups in advanced NSCLC patients treated with nivolumab and durvalumab monotherapy.

## Introduction

Lung cancer is the cause of the highest carcinoma-associated mortality among all malignancies (1). Programmed death receptor-1/programmed death ligand-1 (PD-1/PD-L1) inhibitors have been approved as standard treatments for non-small cell lung cancer (NSCLC) without targeted mutations based on studies showing improved survival over chemotherapy (2–7). These agents are antibodies that induce reactivation of the immune system to target tumor cells by blocking PD-1 or PD-L1 and change the tumor environment by activating tumor-reactive cytotoxic T lymphocytes (CTLs) (8). Despite the significant responses to immune checkpoint inhibitors (ICIs) demonstrated by many trials, the durable clinical benefit rate is only ~20% in a biomarker-selected cohort treated with single agents (2–4, 9). Given the increasing use of ICIs, there is an urgent need to investigate factors that may predict both response and prognosis. Most biomarkers have covered the tumor microenvironment. PD-L1 immunohistochemical expression is a well-studied biomarker in ICIs and has been proven by the American Food and Drug Administration (FDA), but there are still confounding testing methods or parameters (10–12). In addition, circulating blood cells, especially mononuclear leukocytes and cytokines, can indirectly reflect the physical immune reaction. To date, studies on melanoma have suggested the predictive ability of peripheral biomarkers in ICI treatment (12–14). This evidence showed that regular peripheral indicators in blood samples are much more cost-effective and easier for clinical application.

Potential peripheral blood PD-1 treatment biomarkers have been proposed in lung cancer cohorts, such as lactate dehydrogenase (LDH), neutrophil-to-lymphocyte ratio (NLR), and platelet-to-lymphocyte ratio (PLR) (15–20). In fact, the innate differences between PD-1 and PD-L1 inhibitors are structural and functional. In comparison with anti-PD-1 therapy, identifying candidates that could predict benefit from PD-L1 inhibitors is also urgent and important. To our knowledge, no research has reported that routine clinical peripheral blood biomarkers are correlated with prognosis for anti-PD-L1 first-line monotherapy. In addition, previous studies focused on the predictive role of baseline hematological indicators; however, blood biomarkers change dynamically with ICI treatment and early clinical response. Whether those biomarkers tested before the fifth dose of ICI treatment showed predictive and prognostic effects remains unknown. Here, we recruited a retrospective cohort to evaluate peripheral blood markers before and after ICI treatment to better characterize patients who would benefit from nivolumab or durvalumab monotherapy.

## Materials and Methods

### Patients and Treatment

Patients diagnosed with unresectable and advanced non-small cell lung cancer from April 2016 to June 2019 in Hunan Cancer Hospital (the Affiliated Cancer Hospital of Xiangya Medical School, Central South University) were reviewed. We retrospectively included patients treated with at least one dose of nivolumab or durvalumab monotherapy and had available data on selected blood cell counts, serum levels of albumin, and LDH tested 0–28 days before the first ICI dose and fifth ICI dose of treatment with nivolumab or durvalumab. Nivolumab was administered every 2 weeks on day 1 as a cycle with 3 mg/kg body weight. Durvalumab was administered every 4 weeks on day 1 as a cycle with 20 mg/kg body weight. In both cohorts, treatment will be terminated for unmanageable toxic effects or disease progression (according to RECIST1.1 criteria). Clinical data including epidemiological information, regimen, evaluation, and follow-up data were collected. The definition of positive PD-L1 expression in tumors is that the number of membrane-stained tumor cells is ≥1% in more than 100 evaluable tumor cells in formalin-fixed paraffin-embedded (FFPE) slides. In the nivolumab cohort, PD-L1 expression was evaluated using the 22C3 PD-L1 immunohistochemistry assay (Dako Medical System). In the durvalumab cohort, PD-L1 expression on tumor cells was evaluated with the SP263 PD-L1 immunohistochemistry assay (Ventena Medical System) (21). The investigation was conducted according to protocols approved by the institutional review board of Hunan Cancer Hospital.

### Study Design

The analysis of the discovery cohort (nivolumab patients) aimed to identify prognostic factor candidates from 14 routine blood markers (albumin, LDH, absolute or relative cell counts of neutrophils, lymphocytes, monocytes, eosinophils, and basophils, etc.). The timepoint of analysis, mentioned as before the first or fifth dose of ICIs, is the 1 or 2 days before the first or fifth cycle ICI treatment. Promising markers (biomarkers with a *P* < 0.05 in the univariate analysis) and optimal cutoff points of all continuous variates were systematically decided by time-dependent receiver operating characteristic (ROC) curve analysis. The response was considered the best clinical response according to RECIST 1.1. Furthermore, progression-free survival (PFS) and overall survival (OS) differences within the discovery cohort were analyzed based on univariate and multivariate Cox (clinical characteristics included) regression survival analytic models. The analysis of an additional validation cohort (durvalumab monotherapy) finally aimed to validate the prediction value of candidate factors for the clinical outcome of durvalumab first-line monotherapy.

### Statistical Analysis

The study measured the difference between promising markers (peripheral blood parameters and PD-L1 expression) and considered best response and clinical benefit using the chi-square test. Kaplan–Meier analysis of PFS and OS was conducted based on these thresholds, with differences between groups separated by biomarkers assessed with the log-rank test. Biomarkers with a *P* < 0.05 in the univariate analysis were included in the multivariate analysis. Clinically significant features, including gender, age, smoking history, Eastern Cooperative Oncology Group (ECOG) score, histology, and lines of treatment were included as covariates in multivariate Cox regression analysis. The data are shown as hazard ratios (HRs) and 95% confidence intervals (CIs). All statistical analyses were conducted with SPSS Version 22 (SPSS Inc., Munich, Germany).

## Results

### Patient Characteristics

In this 76-patient cohort, 59 patients were treated with nivolumab as first-line or second-line immunotherapy, while 17 patients received durvalumab for first-line immunotherapy. Approximately half of these patients were diagnosed with adenocarcinoma (55.3%), and 78.9% of patients were former or active smokers, while a minority were never smokers (21.1%) (Table 1). Thirty-two (42.1%) patients were positive for (had immunohistochemistry test of) PD-L1 expression, 10 were negative for PD-L1 expression, and 34 patients were unknown for PD-L1 expression (Table 2). All patients had peripheral test results before the first and fifth doses of ICI treatment. The median follow-up period was 7.1 months (range, 0.5–38.5 months) (data collection lock time: June 2019); thus far, 59 (77.6%) patients had progressed disease or died of cancer, and 39 (51.3%) patients are still under follow-up. Among patients who received nivolumab, the median PFS and OS were 6.23 and 8.47 months, respectively. For the durvalumab cohort, the median PFS and OS were 5.40 and 14.5 months, respectively (Supplementary Figure 1). For the 68 patients that had the best response assessment, the SD-PR group comprised 38 patients with 17 patients with partial response (PR) and 21 patients with stable disease (SD), while the PD group comprised 30 patients. Eight patients received <4 weeks of treatment or did not have a response assessment (Table 2).

**Table 1:** Clinical characteristics of patients included.

**Parameters**	**Total (*N* = 76)**	**Nivolumab (*N* = 59)**	**Durvalumab (*N* = 17)**
	***N* (%)**	***N* (%)**	***N* (%)**
**Age (years)**			
Median	61	62	60
Range	35–74	36–74	35–72
**Gender**			
Female	10 (13.2)	8 (13.6)	2 (11.8)
Male	66 (86.8)	51 (86.4)	15 (88.2)
**Histology**			
Adenocarcinoma	42 (55.3)	32 (54.2)	10 (58.8)
Squamous cell	34 (44.7)	27 (45.8)	7 (41.2)
TNM staging^*^			
III	11 (14.5)	11 (18.6)	0 (0)
IV	65 (85.5)	48 (81.4)	17 (100)
**ECOG score**			
0–1	69 (90.8)	52 (88.1)	17 (100)
2	7 (9.2)	7 (11.9)	0 (0)
**Smoking history**			
Yes	60 (78.9)	48 (81.4)	12 (70.6)
No	16 (21.1)	11 (18.6)	5 (29.4)
**Line of treatment**			
First	20 (26.3)	3 (5.1)	17 (100)
Second	56 (73.7)	56 (94.9)	0 (0)

* *Based on the AJCC 8th edition*.

**Table 2:** Markers and the best clinical response in the merged cohort.

**Parameters**	**PD**	**SD-PR**	**HR (95% CI)**	***p*-value**
	**Patients with response data (*****N*** **=** **68)**			
PLR-H (>168.13)	17	10	3.662 (1.318–10.170)	0.0111^*^
PLR-L (≤ 168.13)	13	28		
Albumin-H (>43 g/L)	9	21	0.347 (0.126–0.952)	0.0372^*^
Albumin-L (≤ 43 g/L)	21	17		
	**Patients with response data and PD-L1 test (*****N*** **=** **42)**			
PD-L1 positive (TPS>1%)	15	17	0.588 (0.139–2.491)	0.4687
PD-L1 negative (TPS>1%)	6	4		

* *Statistically significant parameters: P < 0.05*.

### Identification of Prognostic Factors Before Treatment in the Nivolumab Monotherapy Cohort

To identify peripheral blood parameters for prognosis for NSCLC patients treated with nivolumab, 14 continuous variables (albumin, LDH, or blood cell counts, etc.) before ICI treatment along with age, gender, histology, ECOG score, smoking status, and lines of treatment were investigated in all patients (*n* = 59) by univariate Cox regression analysis (Supplementary Table 1). Factors significantly positively correlated with PFS and OS were high albumin (for PFS, *P* = 0.013; for OS, *P* = 0.001) and high absolute lymphocyte counts (for PFS, *P* = 0.041; for OS, *P* = 0.052). Factors that had a significantly negative correlation with PFS and OS were high NLR (for PFS, *P* = 0.01; for OS, *P* = 0.005) and high PLR (for PFS, *P* = 0.043; for OS, *P* = 0.018). No statistically significant correlation between other factors (*P* > 0.05) and PFS or OS was observed in the survival analysis (Supplementary Table 1).

### Categorization of PLR and Albumin Before Treatment and Differentiation of the Best Response

**Figure 1:**
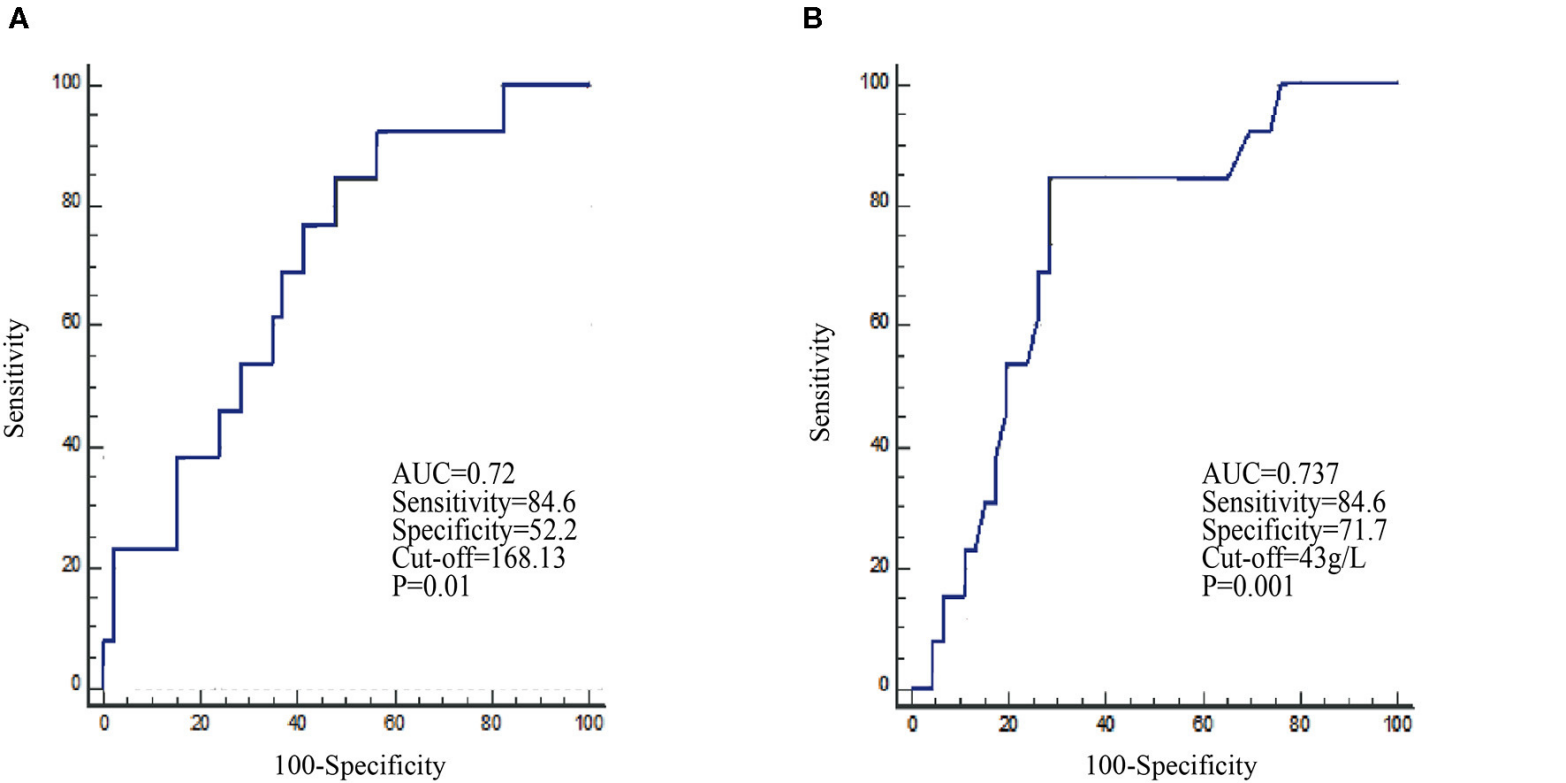
Receiver operating characteristic (ROC) curves identify the baseline PLR **(A)** and albumin **(B)** levels in the prediction of the 11-month survival rate in the nivolumab cohort.

To facilitate possible ultimate clinical utility and verify whether patients with good efficacy can be distinguished, PLR and albumin levels before ICI treatment were dichotomized into two groups based on the prediction of the 11-month death rate. ROC curves were used to determine the optimal cutoffs for survival. For PLR and albumin levels, the AUC was 0.702 (*P* = 0.01, Youden index = 0.3679) and 0.737 (*P* = 0.001, Youden index = 0.5635), respectively (Figure 1). The optimal cutoffs of PLR and albumin, as determined by AUC, were 168.13 and 43 g/L, respectively. For NLR and absolute lymphocytes, we did not observe any statistical significance in the ROC curve analysis (Supplementary Figure 2).

**Figure 2:**
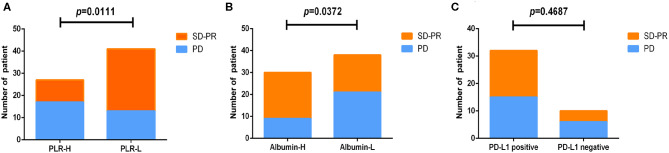
Analysis of markers before treatment and the best response to nivolumab or durvalumab monotherapy. All patients were divided into two groups according to PLR **(A)**, albumin **(B)**, and PD-L1 **(C)** expression before treatment.

To study blood markers before treatment that could be used as predictors of best clinical response, we analyzed PLR, albumin, and PD-L1 expression using chi-square analysis (Table 2). Among the results from PLR, the PLR-H (>168.13) group had an inferior SD/PR rate than the PLR-L (≤ 168.13) group did (10/27, 37.0% vs. 28/41, 68.3%, HR = 3.662, 95% CI: 1.318–10.17, *P* = 0.011) (Figure 2A). For albumin, the SD/PR rate was higher in the albumin-H group (>43 g/L) than in the albumin-L group (≤ 43 g/L) (21/30, 70% vs. 17/38, 44.7%, HR =0.347, 95% CI: 0.126–0.952, *P* = 0.0372) (Figure 2B). However, we did not find significant differences in the best clinical response between PD-L1-positive and PD-L1-negative patients (Figure 2C). From the above, we concluded that PLR and albumin are probably better predictive markers to differentiate the best response of ICIs than PD-L1 expression.

### PLR and Albumin Before Treatment Related to Survival in the Nivolumab Monotherapy Cohort

**Figure 3:**
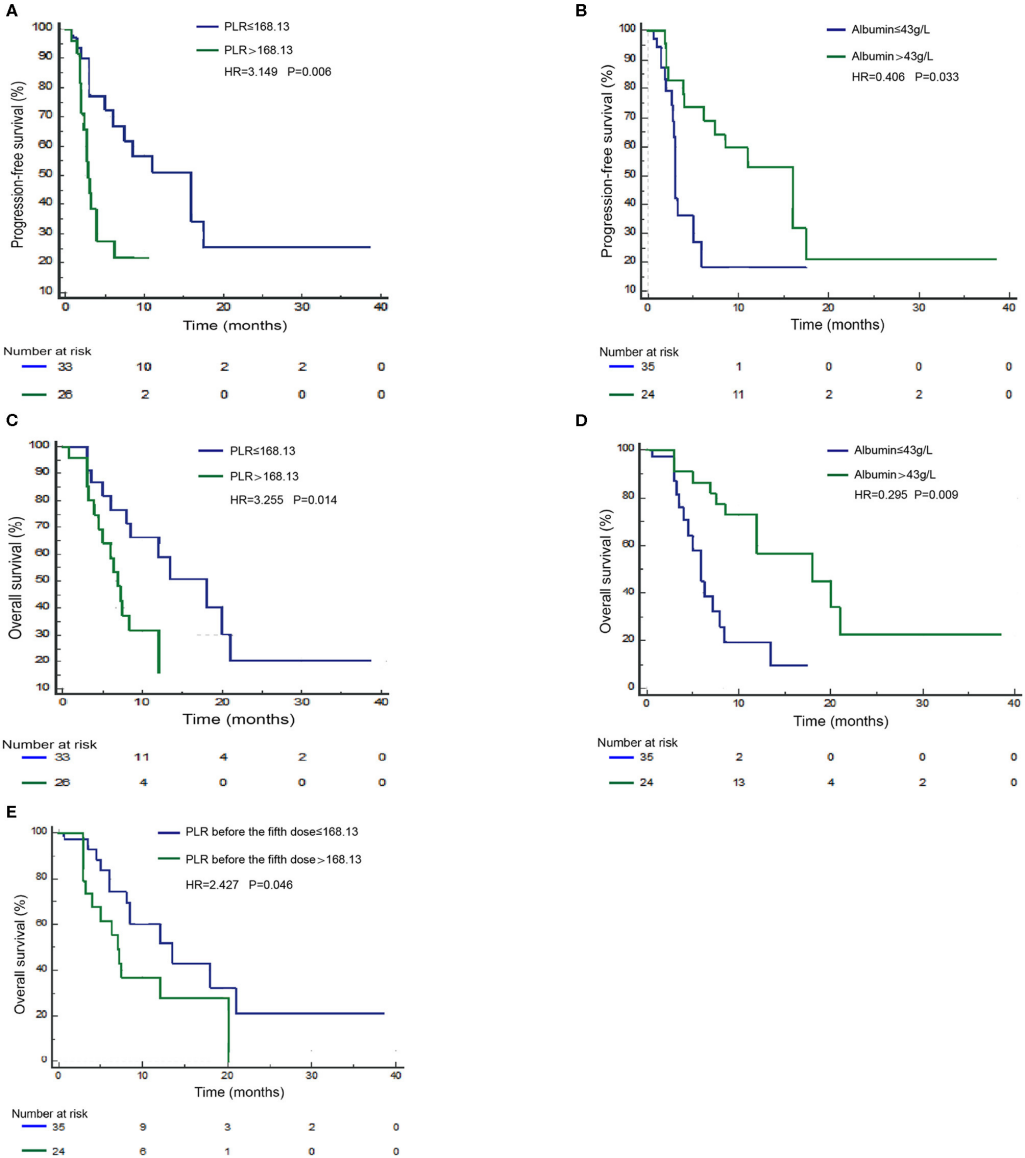
Survival curves under biomarker-defined subgroups. Kaplan–Meier survival curves of progression-free survival (PFS; **A**) and overall survival (OS; **C**) of nivolumab-treated patients were stratified by the platelet-to-lymphocyte ratio (PLR) before ICI treatment. PFS **(B)** and OS **(D)** of nivolumab-treated patients were stratified by albumin before treatment. OS **(E)** of nivolumab-treated patients was stratified by PLR before the fifth dose.

Next, we validated the defined cutoff points of PLR [ ≤ 168.13 (*n* = 33) vs. >168.13 (*n* = 26)] and albumin [ ≤ 43 g/L (*n* = 35) vs. >43 g/L (*n* = 24)] in the nivolumab cohort. Two factors were again significantly associated with response and prognosis by both univariate and multivariate Cox regression survival analysis. Univariate analysis of two factors found that albumin ≥43 g/L before first ICI treatment was related to improved PFS and OS, and PLR ≥168.13 before first ICI treatment was evidently associated with worse PFS and OS (Supplementary Table 2). Since previous studies reported that age, gender, ECOG score, smoking status, and lines of treatment are factors that influence the survival of ICI treatment (15–20), we then performed multivariate Cox proportional regression analysis including these covariates to identify independent factors. The associations between albumin, PLR, and prognosis were again significant in multivariate analysis (Table 3) for PLR and PFS (Figure 3A), HR = 3.149, 95% CI: 1.395–7.413, *P* = 0.006; for albumin and PFS (Figure 3B), HR = 0.406, 95% CI: 0.174–0.931, *P* = 0.033; for PLR and OS (Figure 3C), HR = 3.255, 95% CI: 1.298–8.802, *P* = 0.014; and for albumin and OS (Figure 3D), HR = 0.295, 95% CI: 0.113–0.727, *P* = 0.009. Therefore, PLR and albumin before first-doze ICI treatment could be used to predict the effect and prognosis in NSCLC patients treated with nivolumab.

**Table 3:** Multivariate survival analysis of PLR and albumin before treatment in the nivolumab cohort.

**Factors**	**Categorization**	**PFS**		**OS**	
		**HR (95% CI)**	***P*-value**	**HR (95% CI)**	***P*-value**
		**PLR and covariates**			
Gender	Male vs. female	0.462 (0.044–4.797)	0.496	0.109 (0.003–1.988)	0.147
Age	Continuous	0.499 (0.220–1.148)	0.096	0.486 (0.207–1.147)	0.094
ECOG PS	0 or 1 vs. 2	1.433 (0.610–3.294)	0.404	1.233 (0.468–3.133)	0.668
Smoking history	Never vs. ever	0.671 (0.165–4.600)	0.623	0.307 (0.064–2.286)	0.178
Line of treatment	First vs. second	3.336 (0.641–61.483)	0.252	*N* (1.108–NR)	0.993
PLR	≤ 168.13 vs. >168.13	3.149 (1.395–7.413)	0.006^*^	3.255 (1.298–8.802)	0.014^*^
		**Albumin and covariates**			
Gender	Male vs. female	0.613 (0.068–5.588)	0.642	0.293 (0.011–4.257)	0.378
Age	Continuous	0.506 (0.227–1.141)	0.095	0.598 (0.260–1.391)	0.224
ECOG PS	0 or 1 vs. 2	1.454 (0.630–3.320)	0.379	1.177 (0.452–2.998)	0.736
Smoking history	Never vs. ever	0.895 (0.229–5.964)	0.889	0.407 (0.089–2.944)	0.292
Line of treatment	First vs. second	1.757 (0.328–32.591)	0.595	NR (0.404–NR)	0.994
Albumin	≤ 43 vs. >43 g/L	0.406 (0.174–0.931)	0.033^*^	0.295 (0.113–0.727)	0.009^*^

* *Statistically significant parameters: P < 0.05*.

### PLR Level After Treatment Predicts Overall Survival in the Nivolumab Monotherapy Cohort

Since PLR and albumin are dynamic markers changing with the immune response as well as anticancer treatment, we assume that the level of these two markers before the fifth dose of ICIs could better define the ICI benefit patients. Therefore, we validated the independent potential markers after treatment with a previously defined cutoff, PLR [ ≤ 168.13 (*n* = 23) vs. >168.13 (*n* = 24)] and albumin [ ≤ 43 g/L (*n* = 27) vs. >43 g/L (*n* = 18)], in NSCLC patients treated with nivolumab. The multivariate analysis revealed that a higher PLR after treatment (before the fifth dose) was significantly correlated with shorter OS (HR = 2.427, 95% CI: 1.016–5.939, *P* = 0.046) (Figure 3E). However, we did not observe a statistical relationship between albumin before the fifth dose and survival. Therefore, the PLR level before the fifth dose could also predict the prognosis of nivolumab (Supplementary Table 3).

### PLR and Albumin After Treatment Correlated With Survival in the First-Line Durvalumab Monotherapy Cohort

**Figure 4:**
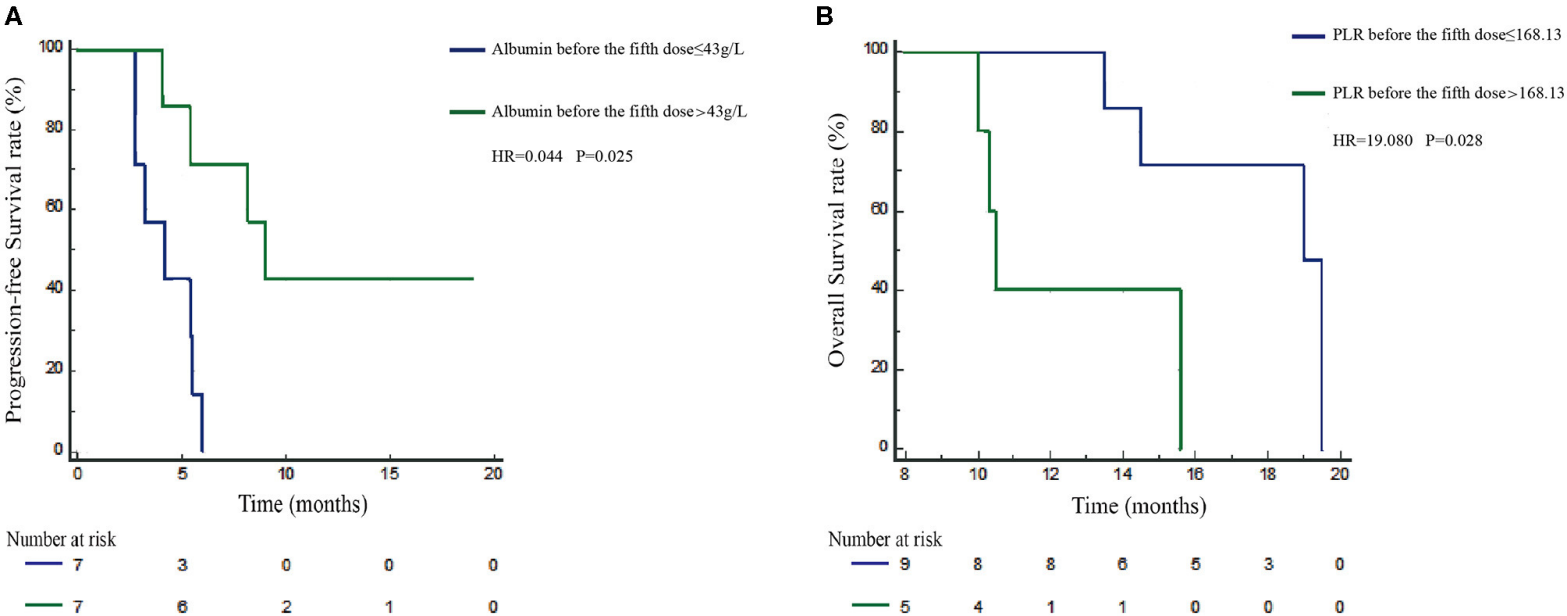
Prognosis validation of PLR and albumin defined subgroups in the durvalumab cohort. Kaplan–Meier survival curves of progression-free survival (PFS; **A**) of durvalumab-treated patients stratified by albumin before the fifth dose. Overall survival (OS; **B**) of durvalumab-treated patients was stratified by PLR before the fifth dose.

To date, the predictive or prognostic value of pre- or post-treatment blood laboratory parameters in NSCLC patients receiving durvalumab monotherapy remains unknown. Due to differences in PD-1 and PD-L1 inhibition mechanisms, we planned to validate the clinical application value of PLR and albumin in NSCLC patients receiving durvalumab. Here, we applied the mentioned cutoff value in the nivolumab cohort to the durvalumab cohort: PLR high (>168.13, *n* = 5) vs. low (≤ 168.13, *n* = 9) and albumin high (>43 g/L, *n* = 7) vs. low (≤ 43 g/L, *n* = 7) (Table 4). We found that albumin high (>43 g/L) before the fifth dose was only significantly related to longer PFS (HR = 0.044, 95% CI: 0.002–0.441, *P* = 0.025) (Figure 4A) but not OS (HR = 3.067, 95% CI: 0.223–33.7, *P* = 0.352). However, a high PLR (>168.13) before the fifth dose was obviously associated with worse OS (HR = 19.080, 95% CI: 1.908–513.115, *P* = 0.028) in multivariate analysis (Figure 4B), and the correlation with OS was consistent with the nivolumab cohort. In addition, we did not observe a significant difference between PLR or albumin before ICI treatment and survival in the durvalumab monotherapy cohort. Hence, PLR may be a prognostic biomarker, and albumin before the fifth dose could be a better indicator for the response of durvalumab.

**Table 4:** Multivariate survival analysis of PLR and albumin before the fifth dose in the durvalumab cohort.

**Factors**	**Categorization**	**PFS**		**OS**	
		**HR (95% CI)**	***P*-value**	**HR (95% CI)**	***P*-value**
		**PLR and covariates**			
Gender	Male vs. female	0.059 (0.002–1.292)	0.091	6.941 (0.078–756.340)	0.376
Smoking history	Never vs. ever	0.088 (0.006–1.007)	0.057	3.55 (0.106–144.091)	0.447
Histology	Squamous vs. adenocarcinoma	0.409 (0.050–2.418)	0.343	0.988 (0.043–12.977)	0.993
PLR	≤ 168.13 vs. >168.13	0.449 (0.078–2.084)	0.323	19.08 (1.908–513.115)	0.028^*^
		**Albumin and covariates**			
Gender	Male vs. female	4.979 (0.055–884.279)	0.52	0.093 (0–18.857)	0.359
Smoking history	Never vs. ever	0.728 (0.050–9.731)	0.81	0.213 (0.006–4.466)	0.324
Histology	Squamous vs. adenocarcinoma	4.389 (0.304–78.056)	0.287	0.197 (0.006–3.413)	0.283
Albumin	≤ 43 vs. >43 g/L	0.044 (0.002–0.441)	0.025^*^	3.067 (0.223–33.700)	0.352

* *Statistically significant parameters: P < 0.05*.

## Discussion

The therapeutic profile for advanced NSCLC has vastly changed for the application of immune checkpoint antibodies. Nivolumab and durvalumab have been approved as standard agents for advanced NSCLC based on studies showing improved OS (2, 3, 5). Despite its superiority to standard chemotherapy, low response rates (2–4, 9) and the clinical usability of new remedy options demand impersonal markers to distinguish patients who benefit from precise immunotherapy. The advantages of routine blood sampling of tumor tissue as a source of biomarkers are that they are easier to test and monitor. Baseline blood cell counts, such as neutrophils, lymphocytes, and NLR, have been associated with response and prognosis in NSCLC and melanoma patients receiving immunotherapy (15–20). We found that PLR and albumin could better help differentiate SD-PR and PD response groups than PD-L1 expression. This study confirmed the association of both increased PFS and OS in the nivolumab cohort with elevated albumin and reduced PLR before ICI treatment as described previously (22, 23). However, potential blood markers for durvalumab first-line monotherapy in NSCLC are still unclear. To date, the present study on NSCLC is the first to show that PLR during treatment (before the fifth dose) is also an independent potential biomarker for OS in both a nivolumab cohort and a durvalumab cohort. Furthermore, albumin levels before the fifth dose are predictive indicators of PFS in NSCLC patients who received first-line durvalumab monotherapy.

PLR, a marker of inflammation, has been investigated in NSCLC accepted PD-1 inhibitors and has been indicated to be an independent negative indicator of survival (15–20, 24, 25). In melanoma, this factor has a potential prognostic effect on patients treated with PD-1 inhibitors (13, 14, 26). Taken together, all previous studies indicate that the prognostic relativity of PLR excludes its predictive value. Inflammation can provide the tumor microenvironment with the materials of inflammatory processes, such as lymphocytes, which can be available biomarkers. They are believed to play a dominant and crucial role in the reaction of ICIs by redistributing lymphocyte subsets from blood to tumor sites (27–29). The results further confirmed that low PLR before ICI treatment might be a significant predictive and prognostic factor for better survival by nivolumab. Furthermore, our data also revealed that high PLR before the fifth dose was significantly associated with worse OS in the patients who received nivolumab and durvalumab monotherapy, whereas it was not associated with PFS which, may indicate that the PLR is prognostic, rather than predictive.

Second, our study discovered that higher albumin before ICI treatment was correlated with better survival in advanced NSCLC patients who received nivolumab, in accordance with a few studies showing that albumin levels may impact the response of NSCLC treated with immunotherapy (22, 23). Serum albumin is a part of the tumor microenvironment and plays a key role in the response to immunological agents. Immunotherapy seemed to speed up the recovery of serum albumin, and the improvement of hepatic functional status expresses the improvement of the tumor microenvironment (30). Hence, for durvalumab first-line monotherapy, we found that higher albumin levels before the fifth dose were significantly associated with longer PFS, which indicates that albumin may be a predictive factor of response. To the best of our knowledge, this is the first study to report the predictive value of albumin in solid tumor patients receiving durvalumab first-line monotherapy. The potential biological mechanism of these findings might contribute to fully understanding and exploring the worth of these biomarkers.

There are several inherent limitations. First, this is a retrospective study with subjective selection bias, so we included covariates that may have the possibility to confound the results. Second, the study lacks a no-ICI group to illustrate whether PLR and albumin are common prognostic factors or predictive factors for ICIs specifically. However, prior investigations reported that the thresholds of NSCLC patients who accepted ICIs were 43.8 g/L and 169–262, respectively (16, 23). The cutoffs found in our research were 43 g/L for albumin and 168.13 for PLR, which is numerically close to the value previously reported. Third, PD-L1 was not required and was available in all patients included. PD-L1 expression was not a necessary test before second-line nivolumab treatment of lung cancer according to guidelines, so PD-L1 expression data were not available in most patients from the nivolumab cohort. Finally, given that this was a single-centre investigation, the cohort was undersized. However, with many cofactors in this cohort, the identified clinical significance was meaningful, and these practicable cutoffs for albumin and PLR require validation in additional cohorts. Since current biomarkers have high false-negative effects, further evaluation of these indicators may be included together with approved indicators in the selection of candidates for immunotherapy.

In conclusion, PLR and albumin could better help differentiate SD-PR and PD response groups than PD-L1 expression. High PLR and low albumin levels are promising predictive or prognostic biomarkers of nivolumab and durvalumab treatment in NSCLC. These markers are easy to dynamically acquire, and are readily available to offer, additional information before or even during treatment. In the future, studies on peripheral blood markers within a prospective independent cohort are needed to further validate the results, which may shed light on how to use those markers to guide and improve first-line ICI monotherapy in NSCLC patients.

## Data Availability Statement

All datasets generated for this study are included in the article/Supplementary Material.

## Ethics Statement

The studies involving human participants were reviewed and approved by the institutional review board of Hunan Cancer Hospital. Written informed consent for participation was not required for this study in accordance with the national legislation and the institutional requirements.

## Author Contributions

LW, MJ, and WP: conception and design. MJ, WP, XP, BC, JL, FX, LL, LX, YX, JC, QW, KL, JW, and LW: acquisition of data (acquired and managed patients). MJ and WP: analysis and interpretation of data. MJ and WP: writing, review, and/or revision of the manuscript. LW: designed and supervised the study. All authors contributed to the article and approved the submitted version.

### Conflict of Interest

The authors declare that the research was conducted in the absence of any commercial or financial relationships that could be construed as a potential conflict of interest.

## Supplementary Material

Supplementary Figure 1 Progression-free survival (PFS) and overall survival (OS) curves show the cohort of patients treated with nivolumab (PFS, **A**; OS, **C**) or durvalumab (PFS, **B**; OS, **D**).

Supplementary Figure 2 Receiver operating characteristic (ROC) curves identify the baseline NLR **(A)** and ALC **(B)** level in the prediction of the 11-month survival rate in the nivolumab cohort.

Supplementary Table 1 Unvariate analysis of 20 markers and survival in nivolumab cohort.

Supplementary Table 2 Univariate analysis of PLR and albumin before treatment and survival in nivolumab cohort.

Supplementary Table 3 Multivariate survival analysis of PLR and albumin before the fifth dose in nivolumab cohort.
