# Supplementary figures and images for: Peripheral Blood Biomarkers Associated With Outcome in Non-small Cell Lung Cancer Patients Treated With Nivolumab and Durvalumab Monotherapy
Source: Front Oncol. 2020 Jun 30;10:913. doi: 10.3389/fonc.2020.00913 (PMC7339928; doi:10.3389/fonc.2020.00913)

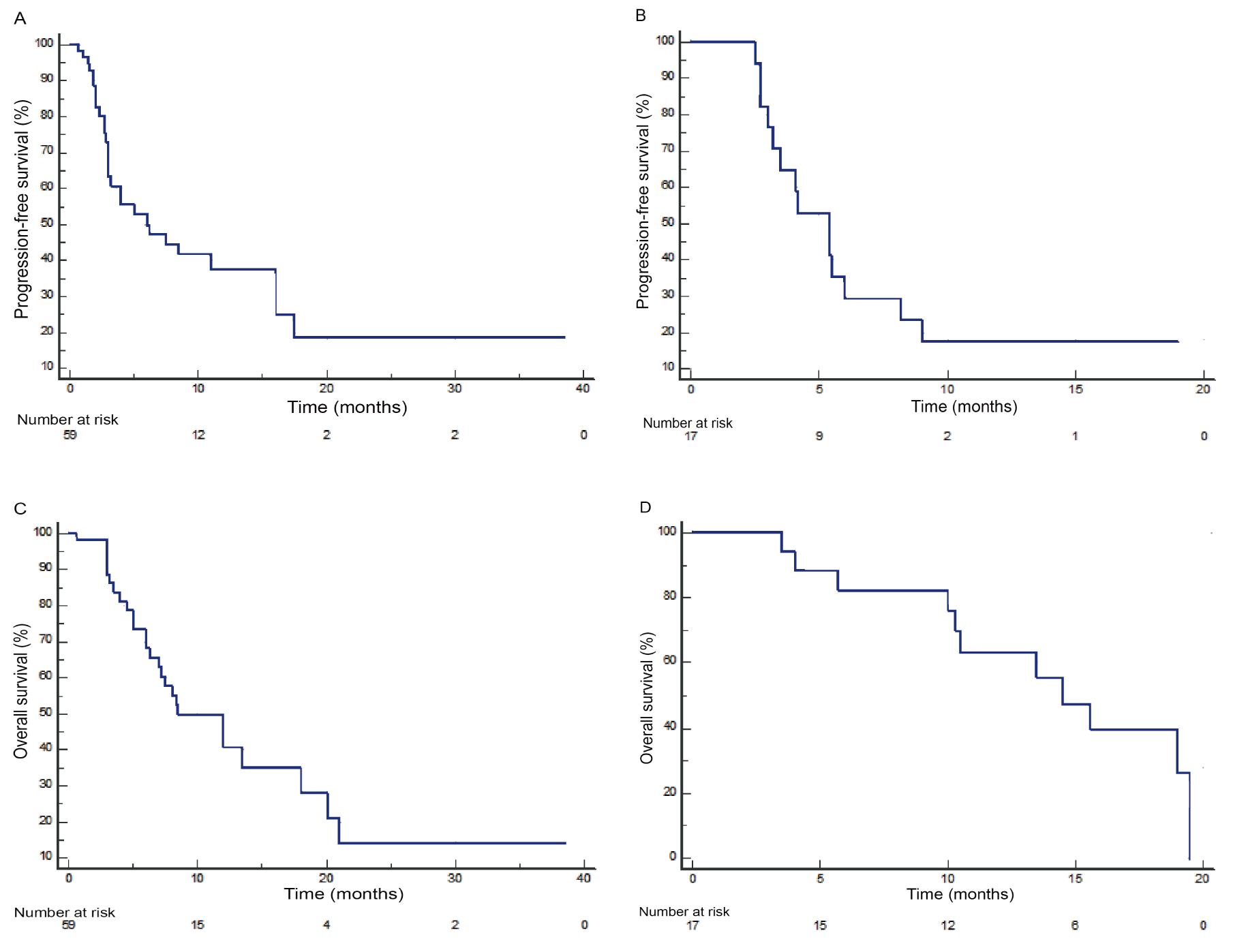

Supplement: Supplementary Figure 1 — Progression-free survival (PFS) and overall survival (OS) curves show the cohort of patients treated with nivolumab (PFS, A; OS, C) or durvalumab (PFS, B; OS, D). [file Image_1.TIF]

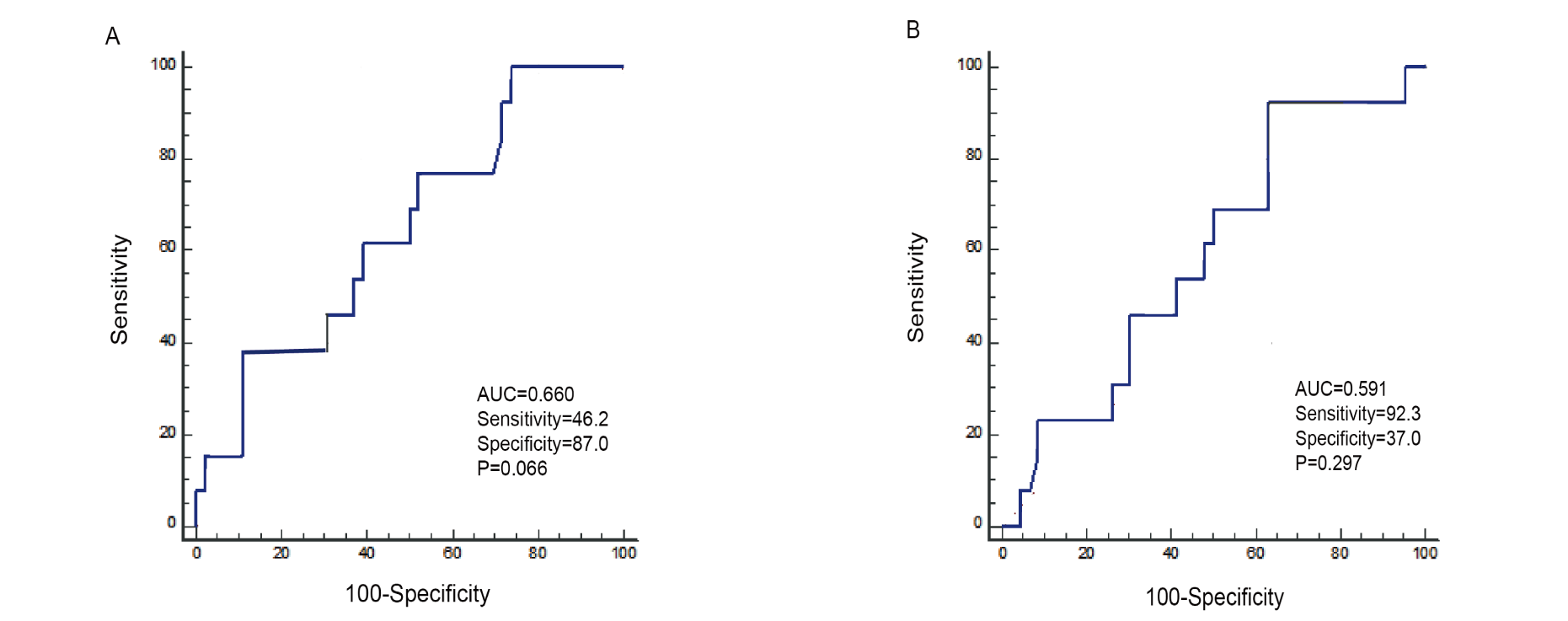

Supplement: Supplementary Figure 2 — Receiver operating characteristic (ROC) curves identify the baseline NLR (A) and ALC (B) level in the prediction of the 11-month survival rate in the nivolumab cohort. [file Image_2.TIF]
