# Supplementary material for: Peripheral Blood Biomarkers Associated With Outcome in Non-small Cell Lung Cancer Patients Treated With Nivolumab and Durvalumab Monotherapy
Source: Front Oncol. 2020 Jun 30;10:913. doi: 10.3389/fonc.2020.00913 (PMC7339928; doi:10.3389/fonc.2020.00913)
Supplement: Supplementary Table 1 — Unvariate analysis of 20 markers and survival in nivolumab cohort. [file Table_1.DOCX]

| Supplementary Table 1. Unvariate analysis of 20 markers and survival in nivolumab cohort | | | | |
| --- | --- | --- | --- | --- |
| Factors | PFS | | OS | |
|  | HR(95% CI) | P-value | HR(95% CI) | P-value |
| Gender | 0.936(0.148-3.275) | 0.930 | 0.763(0.042-3.698) | 0.793 |
| Age | 0.621(0.294-1.330) | 0.211 | 0.706(0.324-1.558) | 0.378 |
| ECOG score | 1.182(0.567-2.463) | 0.657 | 1.232(0.561-2.695) | 0.604 |
| Smoking history | 0.777(0.282-2.730) | 0.655 | 0.573(0.191-2.470) | 0.377 |
| Line of treatment | 1.964(0.413-35.153) | 0.509 | 3685551(0.732-NR) | 0.992 |
| Histology | 0.915(0.435-1.912) | 0.812 | 0.916(0.415-1.995) | 0.826 |
| albumin | 0.918(0.859-0.985) | 0.013* | 0.898(0.842-0.961) | 0.001* |
| LDH | 0.999(0.996-1.001) | 0.480 | 1.000(0.996-1.001) | 0.704 |
| Absolute lymphocytes | 0.537(0.289-0.954) | 0.041* | 0.532(0.273-0.981) | 0.052 |
| Relative lymphocytes | 0.963(0.923-1.000) | 0.063 | 0.961(0.916-1.002) | 0.074 |
| Absolute neutrophils | 0.999(0.902-1.081) | 0.985 | 1.008(0.903-1.095) | 0.865 |
| Relative neutrophils | 1.033(1.000-1.069) | 0.059 | 1.032(0.996-1.073) | 0.092 |
| Absolute monocytes | 0.642(0.167-1.883) | 0.471 | 0.833(0.236-2.224) | 0.747 |
| Relative monocytes | 0.948(0.817-1.060) | 0.424 | 0.972(0.841-1.084) | 0.665 |
| Absolute eosinophils | 1.078(0.663-1.390) | 0.657 | 1.038(0.654-1.328) | 0.818 |
| Relative eosinophils | 0.997(0.903-1.067) | 0.943 | 0.998(0.904-1.068) | 0.966 |
| Absolute basophils | 1.061(0-4880.699) | 0.991 | 0.249(0-1339.507) | 0.796 |
| Relative basophils | 0.684(0.185-2.066) | 0.536 | 0.555(0.131-1.881) | 0.386 |
| PLR | 1.003(1.000-1.005) | 0.043* | 1.004(1.000-1.006) | 0.018* |
| NLR | 1.161(1.026-1.298) | 0.010* | 1.185(1.043-1.336) | 0.005* |

*Statistically significant parameters: P value＜0.05.
