# Supplementary material for: Peripheral Blood Biomarkers Associated With Outcome in Non-small Cell Lung Cancer Patients Treated With Nivolumab and Durvalumab Monotherapy
Source: Front Oncol. 2020 Jun 30;10:913. doi: 10.3389/fonc.2020.00913 (PMC7339928; doi:10.3389/fonc.2020.00913)
Supplement: Supplementary Table 2 — Univariate analysis of PLR and albumin before treatment and survival in nivolumab cohort. [file Table_2.DOCX]

| Supplementary Table 2. Univariate analysis of PLR and albumin before treatment and survival in nivolumab cohort | | | | | | | |
| --- | --- | --- | --- | --- | --- | --- | --- |
| Factors | Categorization | PFS | | | OS | | |
|  |  | HR | 95% CI | P-value | HR | 95% CI | P-value |
| Albumin | ≤43g/L vs >43g/L | 0.396 | 0.177-0.865 | 0.021* | 0.239 | 0.094-0.566 | 0.002* |
| PLR | ≤168.13 vs >168.13 | 3.036 | 1.368-7.030 | 0.007* | 2.851 | 1.204-7.245 | 0.020* |

*Statistically significant parameters: P value<0.05.
