# Supplementary material for: Peripheral Blood Biomarkers Associated With Outcome in Non-small Cell Lung Cancer Patients Treated With Nivolumab and Durvalumab Monotherapy
Source: Front Oncol. 2020 Jun 30;10:913. doi: 10.3389/fonc.2020.00913 (PMC7339928; doi:10.3389/fonc.2020.00913)
Supplement: Supplementary Table 3 — Multivariate survival analysis of PLR and albumin before the fifth dose in nivolumab cohort. [file Table_3.docx]

Table S3. Multivariate survival analysis of PLR and albumin before the fifth dose in nivolumab cohort.

| Factors | Categorization | PFS | | OS | |
| --- | --- | --- | --- | --- | --- |
|  |  | HR(95% CI) | P-value | HR(95% CI) | P-value |
| PLR and covariates | | | | | |
| Gender | male vs. female | 0.305(0.03-3.04) | 0.286 | 0.081(0.003-1.324) | 0.086 |
| Age | continueous | 0.547(0.238-1.267) | 0.153 | 0.533(0.227-1.268) | 0.148 |
| ECOG PS | 0 or 1 vs 2 | 1.65(0.732-3.668) | 0.223 | 1.538(0.635-3.646) | 0.335 |
| Smoking history | no vs. yes | 0.436(0.106-2.956) | 0.304 | 0.169(0.034-1.255) | 0.043* |
| Line of treatment | first vs second | 1.896(0.356-35.094) | 0.545 | NR(0.551-NR) | 0.993 |
| PLR | ≤168.13 vs >168.13 | 2.078(0.903-4.809) | 0.084 | 2.427(1.016-5.939) | 0.046* |
| Albumin and covariates | | | | | |
| Gender | male vs. female | 0.54(0.059-4.962) | 0.561 | 0.251(0.009-3.761) | 0.327 |
| Age | continueous | 0.523(0.233-1.189) | 0.116 | 0.569(0.244-1.343) | 0.19 |
| ECOG PS | 0 or 1 vs. 2 | 1.523(0.657-3.509) | 0.326 | 1.388(0.549-3.468) | 0.487 |
| Smoking history | no vs. yes | 0.651(0.168-4.321) | 0.586 | 0.294(0.063-2.152) | 0.156 |
| Line of treatment | first vs. second | 2.327(0.443-42.939) | 0.423 | NR(0.736-NR) | 0.993 |
| Albumin | ≤43g/L vs >43g/L | 0.513(0.212-1.128) | 0.113 | 0.447(0.172-1.037) | 0.074 |

^*^statistic significant parameters: *P-*value ＜ 0.05.
